# The impact of combined narrative and evidence-based nursing on cancer patients with a peripherally inserted central catheter: A retrospective study

**DOI:** 10.12669/pjms.41.9.12676

**Published:** 2025-09

**Authors:** Juan Hu, Fangfang Weng

**Affiliations:** 1 Juan Hu Department of PICC Clinic, First People’s Hospital of Linping District, Hangzhou, Zhejiang Province 311100, P.R. China; 2 Fangfang Weng Department of Infectious Diseases, First People’s Hospital of Linping District, Hangzhou, Zhejiang Province 311100, P.R. China

**Keywords:** Cancer, Evidence-based nursing, Narrative nursing, Peripherally inserted central Catheter

## Abstract

**Objective:**

To evaluate the impact of narrative nursing combined with evidence-based nursing on the emotional well-being, self-health behavior, quality of life (QOL) and complications in cancer patients with peripherally inserted central catheter (PICC).

**Methods:**

This retrospective study included 119 cancer patients with PICC, treated at the First People’s Hospital of Linping District, Hangzhou, from February 2022 to September 2024. According to the different nursing plans, patients were divided into a narrative (n=62, narrative nursing combined with evidence-based nursing) and a conventional group (n=57, routine nursing). The extent of negative emotions, self-health behaviors, quality of life (QOL) and incidence of complications was assessed in both groups before and after the intervention.

**Results:**

After the intervention, the Self-Rating Depression Scale (SDS) and Self-Rating Anxiety Scale (SAS) scores were reduced in both groups, with more pronounced improvements observed in the narrative group (P<0.05), suggesting a potential association with the combined intervention. Postintervention scores for self-concept, health knowledge, self-responsibility, self-care skills, and SF-36 showed improvement in both groups, with higher scores observed in the narrative group (P<0.05), indicating possible benefits linked to the combined approach. The narrative group also had a lower incidence of complications (11.2%) compared to the conventional group (26.4%) (P<0.05), which may suggest an association with the nursing model.

**Conclusions:**

The combination of narrative and evidence-based nursing can more effectively alleviate the negative emotions of cancer patients with PICC, enhance their self-health behaviors, improve QOL and reduce complications.

## INTRODUCTION

Peripherally inserted central Catheter (PICC) is widely used in cancer patient treatment as it has high vascular selectivity, requires simple nursing care and is characterized by long retention time, low risk of infection, high success rate and minimal trauma.^1^,^2^ However, studies show that if left for a long time, PICC in cancer patients increases the risk of thrombosis, dislodgement, ectopic placement and catheter blockage that negatively impacts both quality of life (QOL) and the effectiveness of primary disease treatment.^3^-^5^ Therefore, providing effective nursing interventions for cancer patients with PICC is crucial. With the evolution of medical and nursing concepts, clinical nursing has shifted from a disease-centered to a patient-centered approach.^6^

Narrative nursing is a psychological intervention measure based on humanistic care that advocates patiently listening to patients’ subjective feelings and providing timely psychological counseling to improve physical and mental state.^7^,^8^ Narrative nursing has been shown to improve negative emotions in lung cancer patients and alleviate their pain levels.^8^ Another approach, evidence-based nursing, also commonly used in the care of cancer patients, combines credible and valuable scientific results with clinical experience and patient needs and develops evidence-based nursing plans.^9^,^10^ The research shows that such approach can improve treatment compliance, lung function, self-efficacy and QOL in lung cancer patients undergoing radiotherapy and chemotherapy.^9^ However, cancer patients with PICC suffer from pain caused by catheter retention and repeated punctures, resulting in poorer psychological conditions compared to ordinary cancer patients.^11^ The effectiveness of evidence-based and narrative nursing approaches on cancer patients with PICC is still unclear, as this group of patients not only requires evidence-based safety care but also specific interventions aimed at reducing the psychological trauma caused by medical devices.^3^-^5^,^9^-^11^

This study retrospectively analyzed the medical records of cancer patients with PICC who received narrative nursing combined with evidence-based nursing at the First People’s Hospital of Linping District, Hangzhou. The study aimed to clarify the impact of the combined nursing approach on negative emotions, self-health behaviors, QOL and complications in this patient group.

## METHODS

This study was designed as a retrospective case-control analysis, based on medical records collected from the First People’s Hospital of Linping District, Hangzhou, involving 119 cancer patients with PICC between February 2022 and September 2024. Group allocation was determined by the nursing protocol applied during different clinical periods. Specifically, patients admitted prior to the implementation of the narrative + evidence-based nursing model received routine care and were categorized into the conventional group, while those treated after the introduction of the new combined nursing protocol formed the narrative group. The intervention was not assigned prospectively, and all data were extracted retrospectively from electronic medical records.

### Ethical approval:

This retrospective study was approved by the Ethics Committee of the First People’s Hospital of Linping District, Hangzhou (Approval No. 2024-059, Date: May 28, 2025). As the study involved only retrospective analysis of anonymized clinical data, the requirement for individual informed consent was formally waived by the ethics committee. All patient information was fully anonymized prior to data extraction and analysis to protect privacy and ensure confidentiality.

### Inclusion Criteria:

- Cancer patients receiving PICC.^1^
- Age of >18 years old.
- The patient’s condition is stable.
- Complete clinical data.

### Exclusion criteria:

- Individuals with consciousness and language barriers.
- Individuals with mental disorders.
- Individuals with organic lesions in organs such as the heart, brain and lungs.
- Individuals with a history of alcohol and drug dependence.
- Patients with PICC placement time of less than six months.
- Patients who died within six months after the intervention.

### Interventions:

### Routine nursing care and health education:

Experienced nursing staff explained the maintenance of PICC catheters and the possible adverse reactions and corresponding treatment measures during catheterization at discharge. A health education manual detailing nursing and preventive measures was distributed. Patients and their families received health education videos on nursing and preventive measures through WeChat chat software. Nurses instructed patients to return to the hospital regularly and on time for follow-up examinations after discharge. During the follow-up period, the responsible nurse accepted patient-initiated phone consultations and proactively conducted telephone follow-ups with discharged patients.

### Narrative nursing:

Prior to the initiation of narrative nursing, all patients were informed that participation was voluntary and that they could withdraw from any session at any time without affecting their clinical care. In addition, nurses involved in the intervention received training in emotional safety and empathetic communication to help them identify and manage potential emotional distress. Throughout the intervention, no patients reported psychological discomfort or refused participation. This suggests good emotional tolerance and acceptability of the intervention within this patient population. To ensure the consistency and fidelity of the narrative nursing intervention, all participating nurses underwent a structured training program before its implementation. The training covered the core principles of narrative nursing, empathetic communication techniques, and emotional safety strategies. A structured interview guide developed by our department was used to standardize the intervention process. This guide consisted of open-ended prompts designed to explore patients’ perceptions of illness, emotional concerns related to PICC, and coping challenges. In addition, nurses participated in supervised simulated interviews to practice the use of the guide and receive feedback. These measures were implemented to minimize variability across sessions and maintain intervention integrity. The approach included several initiatives:

Establishing trust relationships and creating a quiet, comfortable and private environment when interacting with patients for the first time. Nursing personnel communicated with patients in a gentle and friendly manner, actively listened to patients’ feelings, concerns and experiences about tumor diseases and PICC. Patients were encouraged to share their stories, such as changes in their lives after falling ill and their initial impressions of PICC. During the patient’s narration, nurses refrained from interrupting and gave the patient full attention.

Collecting and organizing patient experiences. Multiple in-depth conversations with patients were conducted to gather their experiences and concerns throughout the disease diagnosis and treatment process. This included changes in relationships with family members, expectations for the future, etc., with a focus on situations related to PICC placement, fear during tube placement and the distress of lifestyle changes after tube placement. The patient’s self-told experiences were recorded in detail to identify key emotional nodes and issues. PICC-related complications were assessed according to standardized diagnostic criteria outlined in the hospital’s PICC nursing management protocol, which adheres to national clinical nursing guidelines in China. Complications included phlebitis, catheter displacement, blockage, puncture site bleeding, and infection. The initial screening of complications was performed by responsible bedside nurses during routine catheter maintenance rounds. All suspected events were subsequently confirmed by attending physicians. During retrospective data extraction, two trained senior nurses independently reviewed and classified complication records from electronic medical and nursing documentation. Any discrepancies were resolved through discussion and consensus. Due to the retrospective nature of the study, blinding of evaluators was not applicable; however, predefined criteria and dual-review procedures were employed to ensure data accuracy and consistency.

### Narrative analysis and response:

Patients’ stories were analyzed to identify the underlying emotions, such as anxiety, helplessness and an inferiority complex, due to changes in the body image and the results were used to develop personalized response strategies. For example, patients who expressed dissatisfaction with the appearance changes after PICC can benefit from stories of successful responses from other patients, shared by the nursing staff. Patients were guided to view catheterization from a positive perspective, emphasizing its importance in treatment and its temporary role as a “little helper.” For patients with negative emotions caused by the tumor and PICC placement, nursing personnel make sure to affirm their feelings, providing emotional support and encouragement (“I can understand your current concerns, but you have always been strong and this placement will help you better overcome the disease”).

### Create a new experience together:

In order to ensure consistency and reproducibility, narrative nursing in this study followed a structured schedule. Each patient received approximately one session per week, and each session lasted around 30 to 40 minutes depending on the patient’s emotional status and willingness to share. The intervention period lasted for a total of six weeks, starting from the time of PICC placement. Sessions were conducted face-to-face during hospitalization or through scheduled outpatient visits, telephone calls, or WeChat follow-ups after discharge. All interactions were recorded in the patient’s narrative nursing log for reference and analysis. To quantify patient engagement, two key indicators were tracked throughout the six-week narrative intervention period. The follow-up completion rate was defined as the proportion of patients who completed at least four out of six scheduled interactions. In the narrative group, this rate was 93.5%. In addition, the average number of WeChat responses per patient during the intervention was 6.4 (SD 1.9), reflecting the frequency and intensity of communication. These indicators were derived from nursing logs and WeChat chat records.

### Evidence-based nursing:

### Specific evidence-based questions:

Specific questions were raised based on the patient problems found in narrative nursing, such as the prevention of complications after PICC and the improvement of patient comfort. The examples of the possible questions are: “How to prevent phlebitis in cancer patients with PICC through effective nursing measures?” or “What interventions can improve the psychological comfort of tumor patients with PICC?”,

### Evidence retrieval:

Relevant research literatures were retrieved from authoritative medical databases (such as PubMed, Cochrane Library, CNKI, etc.). The keywords include “PICC”, “tumor patients”, “nursing”, “complication prevention”, etc. The retrieved studies were screened and high-quality research results (such as randomized controlled trials, systematic reviews, etc.) were selected as evidence sources.

### Evidence integration and application:

The evidence was integrated to develop evidence-based nursing measures to enhance the patient’s sense of control and security. Evidence-based measures included an appropriate catheter fixation method to reduce the friction of the catheter to the vascular wall; flushing and sealing methods; effective pain management measures (such as appropriate analgesia methods, including non-drug or drug analgesia, according to the pain degree of patients after catheterization); personalized health education, etc.

Effect evaluation. The nursing effect was evaluated regularly and included the reduced incidence of PICC catheter complications, improved patients’ psychological state (assessed by the psychological scale or self-report). According to the evaluation results, the nursing plan was adjusted and the nursing quality was continuously improved.

### Data collection and outcomes:

### The following indices were analyzed:

- General demographic characteristics and clinical data, including gender, age, education level, marital status, tumor type, tumor stage and treatment.
- *Anxiety and depression*: Self-rating Anxiety Scale (SAS) and Self-rating Depression Scale (SDS) were used to evaluate the patient. Based on the SAS score (maximum 80), patients were diagnosed with severe anxiety (≥ 70), moderate anxiety (60-69), mild anxiety (50-59) and no anxiety (<50). Based on the SDS scores (maximal score of 80 points), patients were diagnosed with severe depression (>72 points), moderate depression (63-72 points), mild depression (53-62 points) and no depression (<53 points).
- *Self-health behavior ability*: The self-care ability scale (ESCA) consisted of 43 items, including self-concept (8 items), health knowledge (17 items), self-care responsibility (6 items) and self-care skills (12 items), with a total score of 172 points. Each item scored 0-4 points. Among them, 11 were reverse scoring entries and the rest were positive scoring entries. A higher score indicated a stronger self-health behavior ability.
- *QOL*: The short form of the Health Survey (SF-36), with a total of 100 points, was used to assess the QOL. The higher score indicated better QOL. 5) Complications, including phlebitis, catheter heterotopia, catheter blockage, bleeding at the puncture point and infection. Complications were evaluated before the intervention and six months after intervention.

### Statistical analysis:

The baseline characteristics of the two groups were compared using a t-test and χ2 test, as appropriate. For measurement data with normal distribution (such as SAS and SDS scores), the results were expressed as mean ± SD; the independent sample t-test was used for comparison between groups and the paired t-test was used for comparison within groups. For skewed data (such as the scores of each sub-item of self-health behavior ability), the results were expressed as median and IQR; the Mann-Whitney U test was used for intergroup comparison and the Wilcoxon signed rank test was used for intragroup comparison. The SF-36 score change curve picture drawing software was GraphPad Prism 7 (GraphPad Software, San Diego, USA). Frequency and constituent ratio (%) were used to describe the counting data and a *χ^2^* test was used. SPSS software version 27.0 (IBM Corp, NY, USA) was used for statistical analysis. All statistical tests were two-sided; P<0.05 was considered statistically significant.

## RESULTS

Ultimately, 119 cases met the study’s inclusion criteria. Among them, 57 patients received routine nursing and 62 patients received narrative nursing combined with evidence-based nursing. There was no significant difference in demographic characteristics and clinical data between the two groups (p>0.05) (Table-I).

**Table-I:** Comparison of demographic characteristics and clinical data between the two groups.

Variables	Narrative group (n=62)	Conventional group (n=57)	χ^2^/t	P
Male (yes), n (%)	37(59.7)	31(54.4)	0.340	0.560
Age (years), mean±SD	54.6±10.3	53.2±11.3	0.715	0.476
Education level, n(%)			0.555	0.456
Junior high school and below	35(56.5)	36(63.2)		
High school and above	27(43.5)	21(36.8)		
Marital status, n(%)			0.486	0.486
In marriage	53(85.5)	46(80.7)		
Out of wedlock	9(14.5)	11(19.3)		
Type of disease, n(%)			4.064	0.255
Lung cancer	24(38.7)	20(35.1)		
Digestive tract neoplasms	19(30.6)	13(22.8)		
Liver cancer	17(27.4)	17(29.8)		
Other types of tumors	2(3.2)	7(12.3)		
Disease stage, n(%)			0.856	0.355
Phase I-II	41(66.1)	33(57.9)		
Phase III-IV	21(33.9)	24(42.1)		
Methods of treatment, n(%)			1.316	0.518
Chemotherapy alone	39(62.9)	38(66.7)		
Combined chemoradiotherapy	11(17.7)	6(10.5)		
Targeted therapy combined with chemotherapy	12(19.4)	13(22.8)		

Before intervention, there was no significant difference in SAS (61.9 ± 6.8 vs. 62.9 ± 7.6) and SDS (62.1 ± 7.8 vs. 63.9 ± 8.3) scores between the narrative group and the conventional group (P>0.05). After intervention, the SAS (41.3 ± 6.4 vs. 48.5 ± 5.4) and SDS (43.3 ± 6.5 vs. 46.5 ± 7.7) scores of the two groups were significantly lower than before the intervention and were substantially lower in the narrative group compared to the conventional group (P<0.05) (Table-II).

**Table-II:** Comparison of negative emotions between the two groups (mean ± SD, scores).

Variables	Narrative group (n=62)	Conventional group (n=57)	t	P
***Before intervention***				
SAS	61.9±6.8	62.9±7.6	-0.703	0.483
SDS	62.1±7.8	63.9±8.3	-1.273	0.206
***After intervention***				
SAS	41.3±6.4^a^	48.5±5.4^a^	-6.56	<0.001
SDS	43.3±6.5^a^	46.5±7.7^a^	-2.437	0.016

Compared with before treatment,

a P<0.05.

In terms of engagement metrics, 58 out of 62 patients (93.5%) in the narrative group completed at least four out of six scheduled WeChat or telephone follow-ups. Furthermore, the mean number of WeChat responses per patient was 6.4 (SD 1.9), indicating a high level of adherence and communication intensity in this group.

Preintervention scores of self-concept, self-responsibility and self-care skills were comparable in both groups (P>0.05). The intervention resulted in a significant increase in these scores in both groups. Postintervention self-concept, self-responsibility and self-care skills scores were considerably higher in the narrative group than in the conventional group (P < 0.05) (Table-III).

**Table-III:** comparison of self-health behavior ability between the two groups (M (IQR), scores).

Variables	Narrative group (n=62)	Conventional group (n=57)	Z	P
***Before intervention***				
Self-concept	17.5 (16-21)	17 (15-20)	-1.062	0.288
Health knowledge	38 (34-42)	39 (37-41)	-1.205	0.228
Self-responsibility	10 (9-12)	11 (9-12)	-1.690	0.091
Self-care skills	19 (18-21)	20 (18-22)	-0.806	0.420
***After intervention***				
Self-concept	23 (21-26)^a^	20 (19-23)^a^	-3.373	<0.001
Health knowledge	54.5 (51-59)^a^	50 (47-53)^a^	-3.978	<0.001
Self-responsibility	17 (16-19)^a^	15 (13-16)^a^	-4.536	<0.001
Self-care skills	25 (24-26)^a^	23 (20-25)^a^	-3.425	<0.001

Compared with before treatment,

a P<0.05.

**Fig.1:**
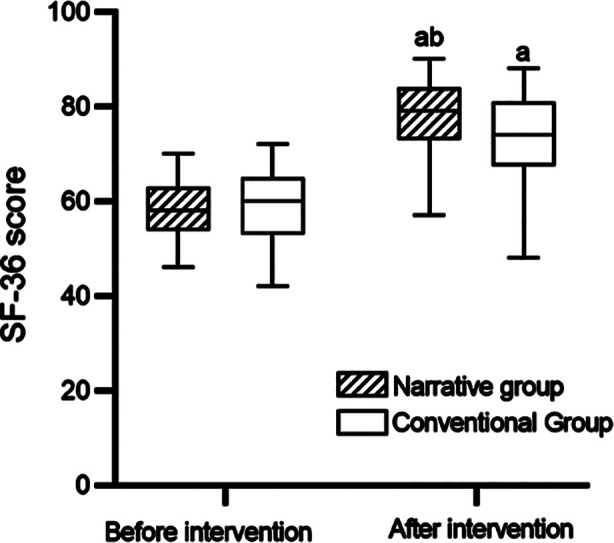
Comparison of SF-36 scores between the two groups. Compared with before treatment, ^a^P<0.05; Compared with the conventional group, ^b^P<0.05.

Before intervention, there was no significant difference in SF-36 score between the groups (58.3 ± 6.4 vs. 59.1 ± 7.9) (P>0.05). After intervention, the SF-36 scores of the two groups increased and were significantly higher in the narrative group (P < 0.05) (Fig.1).

The occurrence of complications is shown in Table-IV. Patients in the narrative group reported two cases of phlebitis, two cases of catheter blockage, one case of puncture point bleeding and two cases of infection, with the total incidence of 11.2% (7/62). In the conventional group, there were four cases of phlebitis, one case of catheter heterotopia, four cases of catheter blockage, three cases of puncture site bleeding and three cases of infection, resulting in a total incidence of 26.4% (15/57) (P < 0.05).

**Table-IV:** Comparison of the incidence of complications between the two groups, n(%).

Complications	Narrative group (n=62)	Conventional group (n=57)	χ^2^	P
Phlebitis	2 (3.2)	4 (7.0)	-	-
Ectopic ductus	0 (0.0)	1 (1.8)	-	-
Catheter blockage	2 (3.2)	4 (7.0)	-	-
Bleeding at puncture point	1 (1.6)	3 (5.3)	-	-
Infected	2 (3.2)	3 (5.3)	-	-
Total incidence	7 (11.2)	15 (26.4)	4.449	0.035

## DISCUSSION

The results of this study suggest that the combination of narrative and evidence-based nursing may be associated with improvements in negative emotions, self-health behaviors, and quality of life (QOL) in cancer patients with PICC. The combined approach was also associated with reduced incidence of complications. Cancer patients with PICC often show varying degrees of anxiety and depression during chemotherapy that, if unaddressed, may develop into somatic symptoms.^3^-^5^ Alleviating the negative emotions of patients is one of the main objectives of a combined narrative/evidence-based nursing approach.^12^-^14^

This study showed that the SAS and SDS scores of the narrative group were significantly lower than those of the conventional group after intervention. These results are consistent with previous research. A study by Sun et al.^12^ demonstrated that narrative nursing care for patients after lung cancer surgery enhanced their social and psychological adaptability and alleviated perioperative symptoms. The study by Xu et al.^13^ demonstrated that narrative nursing effectively reduces negative emotions in patients with malignant tumors, relieves anxiety, enhances treatment confidence and quality of life (QOL) and is associated with high nursing satisfaction.

A multicenter study by Su et al.^14^ further confirmed that evidence-based continuous nursing can improve the self-efficacy, QOL and satisfaction of patients with colorectal cancer. The current study implemented a narrative nursing combined with evidence-based nursing for cancer patients with PICC lines. It demonstrated that the combined approach efficiently alleviated the degree of anxiety and depression of patients, in agreement with the study by Huang et al.^15^ It is plausible that the observed improvement in psychological state is due to the synergistic effect of the two nursing approaches. Narrative nursing enables patients to express their deep feelings and release depressed emotions by listening to the patient’s story and provides targeted emotional support and psychological counseling.^13^-^15^ At the same time, evidence-based nursing uses existing research evidence to provide scientific methods for relieving patients’ negative emotions.^15^

At the six months follow-up, the self-health behavior ability and the SF-36 scores of patients in the narrative group were significantly higher than in the conventional group. This demonstrates that narrative nursing, combined with evidence-based nursing, has significant advantages in enhancing the self-health behavior ability of patients with cancer who have a PICC and is conducive to improving their quality of life.^15^,^16^ Narrative nursing can help patients reshape and enhance their positive self-image.^15^-^17^ At the same time, evidence-based nursing provides the best practice method to improve the level of health knowledge, enhance self-responsibility and self-care skills of patients.^7^,^17^Additionally, the evidence-based health education model enhances patients’ understanding of PICC and improves their sense of responsibility and self-care skills.^16^ Numerous studies have also pointed out that narrative nursing alleviates the psychological pressure of patients and enables them to face the disease and life after catheterization with a more positive attitude.^16^-^18^ Evidence-based nursing can also reduce the incidence of catheter-related complications and effectively improve patients’ quality of life.^18^,^19^

This study showed that the combined approach is associated with a lower rate of complications. Bertoglio et al.^20^ showed that the incidence of complications in patients with chemotherapy-treated tumors after PICC was 24.7%. A study by Kang et al.^21^ that included 477 cancer patients found a complication rate of 17% for PICC. This study showed that the incidence of complications in the narrative group was 11.2%, which was significantly lower than that in the conventional group (26.4%). Narrative nursing combined with evidence-based nursing can, therefore, effectively reduce the incidence of complications in tumor patients during PICC catheter treatment. After discharge, patients receive continuous education on PICC catheter management and care through the WeChat platform, further enhancing their ability to manage emergencies. At the same time, timely interaction with healthcare providers through the WeChat platform reduces discomfort and improves confidence in controlling the disease,^20^-^22^ which ultimately will lower the incidence of complications.

Additionally, the emotional safety and patient tolerance of the narrative intervention were satisfactory. No cases of intervention refusal or early withdrawal due to emotional discomfort were observed. We believe this is likely due to adequate psychological preparation prior to the intervention and the supportive, non-coercive environment created by trained nurses. These findings support the acceptability of the combined narrative and evidence-based approach in emotionally vulnerable populations such as cancer patients with PICC lines.

The observed benefits of the combined narrative and evidence-based nursing intervention may be partially explained by established behavioral and psychological theories. First, the reduction in anxiety and depression symptoms aligns with emotional regulation theory, as narrative nursing facilitates emotional expression, cognitive reframing, and psychological adaptation.^12^ Second, the enhancement of self-care behaviors is consistent with Bandura’s self-efficacy theory, in which personalized education and reinforcement build patients’ confidence in managing health-related challenges.^15^ Third, the reduction in complications and improvement in quality of life may relate to increased health literacy, as evidence-based education enables patients to better understand, evaluate, and apply medical information.^17^,^22^ These theoretical perspectives provide a useful framework to interpret the multi-dimensional effects observed in this study and support the rationale for integrating humanistic and evidence-based approaches in oncology nursing.

This study combines narrative medicine and evidence-based practice of dual-mode nursing, from the traditional nursing mode of “disease-centered” to the concept of “patient-centered”. These findings contribute to the understanding of the potential benefits of combining narrative and evidence-based nursing for cancer patients with PICC and offer preliminary support for its use in clinical practice.

This study offers several meaningful contributions to the existing literature. First, it adds to the limited body of research evaluating the use of combined narrative and evidence-based nursing specifically in cancer patients with PICC lines, highlighting its potential benefits across emotional, behavioral, and clinical outcomes. Second, the findings underscore the clinical relevance of integrating psychological support with technical care, suggesting a shift toward more holistic, patient-centered nursing models in oncology. Third, the study benefits from the use of structured nursing logs, real-world clinical data, and validated multi-dimensional outcome measures, which strengthen the practical value and reliability of the results. Lastly, future research should aim to verify these findings through larger-scale, prospective, and multi-center studies, and explore the mechanisms underlying the observed effects, such as emotional regulation, health literacy, and patient empowerment.

### Limitations:

First, this is a retrospective analysis conducted in only one hospital. Secondly, the long-term effects of this nursing model are not yet apparent. Further studies with longer follow-ups are needed to clarify whether patients’ self-health behavior ability can be maintained. Although the total sample size (n = 119) may be considered relatively small when divided into groups, the consistency of significant findings across multiple outcomes supports the credibility of the observed effects. Given the retrospective nature of the study, we did not perform a post hoc power analysis, which is typically more appropriate for prospective trials with pre-defined sample sizes. However, we acknowledge this as a limitation and suggest that future research should confirm these findings using larger-scale, prospective, and adequately powered designs. Additionally, although the combined nursing mode has shown promising results, individual differences may exist among patients. Future research should investigate how to further optimize this nursing model based on patient age, tumor type, cultural background and other relevant factors and develop more personalized nursing programs tailored to meet the diverse needs of patients. Furthermore, although the study demonstrates the positive impact of narrative combined with evidence-based nursing, the molecular and neurophysiological mechanisms underlying this effect have not been thoroughly explored. Although this study was conducted in a tertiary hospital setting with access to trained staff and digital follow-up systems, the feasibility of implementing the combined nursing approach in primary care or resource-limited settings warrants further consideration. On the one hand, narrative nursing primarily relies on communication and listening skills, making it potentially feasible even in low-resource contexts if appropriate training is provided. On the other hand, challenges such as limited staffing, insufficient training in narrative techniques, and restricted access to digital platforms (e.g., WeChat) may hinder implementation in rural or under-resourced regions. To address these barriers, simplified intervention protocols, standardized communication scripts, and scalable training modules could be developed. Furthermore, leveraging remote nursing technologies and community-based support networks may help extend the reach and sustainability of the combined nursing model in diverse healthcare environments.

## CONCLUSION

The findings suggest that narrative nursing, when combined with evidence-based interventions, may help alleviate negative emotions, support self-health behavior, reduce complications, and potentially improve quality of life (QOL) in cancer patients with PICC. This combined nursing approach offers a more comprehensive and effective method for caring for cancer patients with PICC lines. Future research should further explore how to optimize this nursing model according to the individual differences of different patients, thereby better meeting their needs.

### Authors’ contributions:

**JH:** Study design, literature search and manuscript writing.

**JH and FW:** Data collection, data analysis and interpretation. Critical Review.

**JH:** Manuscript revision and validation and is responsible for the integrity of the study.

All authors have read and approved the final manuscript.
